# Circulating endocannabinoidome signatures of disease activity in amyotrophic lateral sclerosis

**DOI:** 10.1111/ene.16400

**Published:** 2024-08-16

**Authors:** Raffaele Dubbioso, Fabio Arturo Iannotti, Gianmaria Senerchia, Roberta Verde, Valentina Virginia Iuzzolino, Myriam Spisto, Ines Fasolino, Fiore Manganelli, Vincenzo Di Marzo, Fabiana Piscitelli

**Affiliations:** ^1^ Department of Neurosciences, Reproductive Sciences and Odontostomatology University of Naples Federico II Naples Italy; ^2^ Institute of Biomolecular Chemistry—National Research Council (ICB‐CNR) Pozzuoli Italy; ^3^ Institute of Polymers Composites and Biomaterials—National Research Council (IPCB‐CNR) Naples Italy; ^4^ Canada Excellence Research Chair on the Microbiome‐Endocannabinoidme Axis in Metabolic Health, Institut Universitaire de Cardiologie et de Pneumologie de Québec and Institut sur la Nutrition et les Aliments Fonctionnels, Centre NUTRISS Université Laval Quebec City Quebec Canada

**Keywords:** ALS, biomarker, endocannabinoid, inflammation, lipid, precision medicine

## Abstract

**Background and purpose:**

Preclinical studies of amyotrophic lateral sclerosis (ALS) have shown altered endocannabinoid (eCB) signalling that may contribute to the disease. Results from human studies are sparse and inconclusive. The aim of this study was to determine the association between serum levels of eCBs or their congeners, the so‐called endocannabinoidome, and disease status and activity in ALS patients.

**Methods:**

Serum concentrations of 2‐arachidonoylglycerol and *N*‐arachidonoylethanolamine (AEA), and AEA congeners palmitoylethanolamide (PEA), oleoylethanolamide (OEA), eicosapentaenoylethanolamide (EPEA), 2‐docosahexaenoylglycerol (2‐DHG) and docosahexaenoylethanolamide (DHEA) were measured in samples from 65 ALS patients, 32 healthy controls (HCs) and 16 neurological disease controls (NALS). A subset of 46 ALS patients underwent a longitudinal study. Disease activity and progression were correlated with eCB and congener levels.

**Results:**

Most circulating mediators were higher in ALS than HCs (all *p* < 0.001), but not NALS. Across clinical stages, ALS patients showed increased levels of PEA, OEA and EPEA (all *p* < 0.02), which were confirmed by the longitudinal study (all *p* < 0.03). Serum PEA and OEA levels were independent predictors of survival and OEA levels were higher in patients complaining of appetite loss. Cluster analysis revealed two distinct profiles of circulating mediators associated with corresponding patterns of disease activity (severe vs. mild). Patients belonging to the ‘severe’ cluster showed significantly higher levels of OEA and PEA and lower levels of 2‐DHG compared to NALS and HCs.

**Conclusion:**

Circulating endocannabinoidome profiles are indicative of disease activity, thus possibly paving the way to a personalized, rather than a ‘one‐fits‐all’, therapeutic approach targeting the endocannabinoidome.

## INTRODUCTION

Amyotrophic lateral sclerosis (ALS) is a neurodegenerative disease characterized by progressive damage of motor neurons in the motor cortex, the brain stem nuclei and the anterior horns of the spinal cord resulting in progressive muscle weakness and wasting [1]. Alternatively, based on the ‘dying back’ hypothesis, ALS may originate in the peripheral tissue, including the skeletal muscle, where a retrograde signalling cascade leads to motor neuron death [2].

Besides motor neuron degeneration, in recent years several studies have consistently demonstrated extra‐motor involvement, paralleling motor worsening [3, 4, 5]. Amongst non‐motor clinical symptoms, an imbalance of energy homeostasis leads to malnutrition and weight and muscle mass loss [6]. Interestingly, the progressive weight loss is also caused by loss of appetite, both considered indicators of poor prognosis [6]. Regarding ALS aetiology, emerging evidence suggests that neuroinflammation, characterized by activated microglia, astrogliosis and immune cell infiltration, plays a critical role in the pathogenesis and severity of the disease [7, 8].

The endocannabinoid (eCB) system acts as a perfect candidate biomarker for patients with ALS. This system, in fact, plays a crucial role in the regulation of metabolism and eating behaviour, with its receptors (CB1R and CB2R) expressed in the brain areas and peripheral organs regulating energy metabolism, immune reactions, inflammatory responses and many other cell functions [9]. The two major endogenous ligands of CB_1_ and CB_2_ receptors are the endocannabinoids (eCBs) anandamide (AEA) and 2‐arachidonoylglycerol (2‐AG).

Dysfunction of the eCB system is indeed implicated in numerous human diseases including those associated with neuroinflammation, neurodegeneration and traumatic injury [10]. Beside the two major eCBs and their responsive receptors, many other endogenous lipid mediators, receptors and enzymes have been associated, due to their chemical and functional proprieties, to the eCB system. Considering this, the scientific community has started to use the term endocannabinoidome (eCBome) to refer to the expanding eCB system. Numerous preclinical studies show that the eCB system is dysregulated in ALS contributing to the disease course. For instance, superoxide dismutase (SOD1) G93A transgenic mice exhibit downregulation of CB_1_ receptors and increased concentrations of both 2‐AG and AEA in the lumbar spinal cord, possibly as part of an endogenous defence mechanism [11]. Interestingly, in the same model the inhibition of fatty acid amide hydrolase (FAAH) and monoacylglycerol lipase, which are the main degradative enzymes for AEA and 2‐AG respectively, delayed the disease onset and progression and increased the survival of animals [12, 13]. Another study [14] demonstrated that both female and male SOD1 G93A mice showed a marked upregulation in the spinal cord of CB_2_ receptors with no change in CB_1_, whereas *N*‐acyl phosphatidylethanolamine phospholipase D (one of the main enzymes for AEA synthesis) was significantly upregulated in males with no changes in FAAH (AEA degradation) expression [14]. In TDP‐43 transgenic mice, an alternative murine model of ALS, Espejo‐Porras et al. [15] demonstrated a small reduction in FAAH and elevated CB_2_ mRNA expression in the ventral horn of the spinal cord of both male and female mice, without significant changes in eCB levels.

Interestingly, most of the preclinical studies recently reviewed by Iannotti [16] point to CB_2_ receptors as contributors to the progression and severity of ALS, rather than CB_1_ or other components of the eCBome. The only study involving ALS patients reported no difference between circulating eCBs compared to healthy controls, nor any association with disease severity [17].

Therefore, based on these findings, the aim was to analyse circulating eCBs in a clinically well‐defined cohort of ALS patients to search for a potential correlation between the levels of major eCBs and congener molecules and disease progression and severity.

## MATERIALS AND METHODS

### Participants and clinical characterization

This prospective longitudinal study consisted of 113 participants seen at the Department of Neurology or the ALS Centre of the University Federico II of Naples, Italy, between January 2021 and October 2023. Specifically, 65 patients with ALS were recruited (41 men; age 68; interquartile range [IQR] 17.5 years), 16 patients (10 men; age 68.5, IQR 21 years) admitted to the neurological inpatient clinic with a condition included in the differential diagnosis of ALS or with a neurodegenerative disease (NALS) and 32 healthy controls (HCs) (20 men, age 64.5, IQR 12.3 years), recruited amongst the caregivers of patients.

Patients with ALS met a diagnosis of ‘probable’, ‘probable laboratory‐supported’ or ‘definite’ ALS, as per the revised El Escorial criteria [18]. Clinical and respiratory assessment, neuropsychological and eating behaviour evaluation [19, 20, 21, 22, 23, 24, 25] and inclusion/exclusion criteria are described in Data S1.

All patients with ALS underwent mutation screening for the four most common ALS‐causing genes, namely *C9orf72* repeat expansion and mutations of *SOD1*, *TARDBP* and *FUS* genes. Three patients (5%) carried a pathogenic hexanucleotide repeat expansion in *C9orf72*, one (1.5%) in *SOD1* and another (1.5%) in the *TARDBP* gene. Clinical assessment and endocannabinoidome analysis were performed on the whole ALS group (*N* = 65), including sporadic and genetic cases.

Written informed consent was obtained from all subjects according to the Declaration of Helsinki before enrolment in the study. The study protocol was approved by the local Ethics Committee (protocol number 100/17/ES01 and 151/2023).

### Sample collection, endocannabinoid and related molecules analysis

For each participant, peripheral blood was collected between 09.00 and 11.00 am, without fasting.

Forty‐six patients with ALS also underwent longitudinal sample collection, whilst 19 patients did not undergo follow‐up analysis due to accrued physical disability, inability to reach our centre, or the patient's desire to discontinue participation. Details on the analysis of endocannabinoid and related molecules [26] are reported in Data S1.

### Statistical analysis

Demographic and clinical characteristics of the participants were reported as percentages for categorical variables and as median (IQR) for non‐parametric continuous variables. Details regarding group comparison analysis, survival and cluster analyses can be found in Data S1.

## RESULTS

### Participant characteristics

The ALS group did not differ from the HC and NALS groups regarding sex (male 63.1% vs. 62.5% vs. 62.5%, *p* = 0.97) and age (*p* = 0.29). The diagnosis of patients included in the NALS group is available as Data S1. Across clinical stages, ALS patients did not show a significant difference in terms of median age at onset, sex distribution, disease duration and the percentage of bulbar onset patients, whilst patients belonging to the more advanced clinical stages displayed higher values of disease progression rate, Penn Upper Motor Neuron Score total score, percentage of patients with cognitive and/or behavioural impairment, and lower values of forced vital capacity and total Medical Research Council score (see Table 1). The overall median disease duration from onset was 27 months (IQR 32.5), and 27.7% (18/65) of patients had died by October 2023. Finally, all patients recruited into the study were treated only with riluzole at the same dosage (100 mg), as a disease‐modifying therapy.

**TABLE 1:** Demographic and clinical data of ALS patients across King's clinical stages.

	King's clinical stage				*p* value	Total
	1	2	3	4		
Age at testing, years	58.5 (11.8)	70 (22)	69 (21.5)	68.5 (24)	0.409	68 (17.5)
Sex (M/F)	4/5	12/4	15/11	10/4	0.382	41/24
Disease duration since onset, months	22.5 (12.8)	24 (36)	27 (42.5)	40 (39.5)	0.570	27 (32.5)
ALSFRS‐R score	42 (3.8)	34 (8)	26 (12.5)	19 (9.3)	**<0.001**	27 (17.5)
Disease progression rate (ALSFRS‐R points lost/month)	0.4 (0.4)	0.8 (0.9)	1.1 (1)	1 (1.1)	**0.022**	0.8 (1)
Onset (spinal/bulbar)	8/1	13/3	19/7	12/2	0.677	52/13
Percentage of patients with cognitive and/or behavioural impairment	11% (1/9)	38% (6/16)	62% (16/26)	64% (9/14)	**0.03**	32/65
FVC (%)	93.5 (24.5)	85 (42)	70 (53.8)	41.5 (25)	**0.002**	70 (51.5)
BMI (kg/m^2^)	24.7 (4.8)	22.1 (6.6)	23.8 (4.9)	23.6 (3.7)	0.327	23.4 (5.4)
PUMNS total score (max 32), lower is better	12.5 (6.5)	11 (8)	17.5 (8.5)	15 (8.5)	**0.038**	15 (9.5)
MRC total score (max 130), higher is better	126.5 (19.8)	102 (51)	82.5 (46.3)	78 (55.3)	**0.005**	86 (60)
Percentage of patients with loss of appetite	11% (1/9)	56% (9/16)	46% (12/26)	43% (6/14)	0.173	43% (28/65)
Percentage of patients with overeating	22% (2/9)	38% (6/16)	23% (6/26)	36% (5/14)	0.682	29% (19/65)

*Note*: Values are expressed as median (interquartile range) or as percentage (frequencies). Comparisons between the four groups were performed by means of non‐parametric Kruskal–Wallis *U* test; frequencies were compared by means of chi‐squared test. Values in bold type indicate significance *p* < 0.05.

Abbreviations: ALS, amyotrophic lateral sclerosis; ALSFRS‐R, ALS Functional Rating Scale Revised; BMI, body mass index; F, female; FVC, forced vital capacity; M, male; MRC, Medical Research Council; PUMNS, Penn Upper Motor Neuron Score.

### Endocannabinoid and related mediator serum concentrations across groups and ALS clinical stages

**FIGURE 1:**
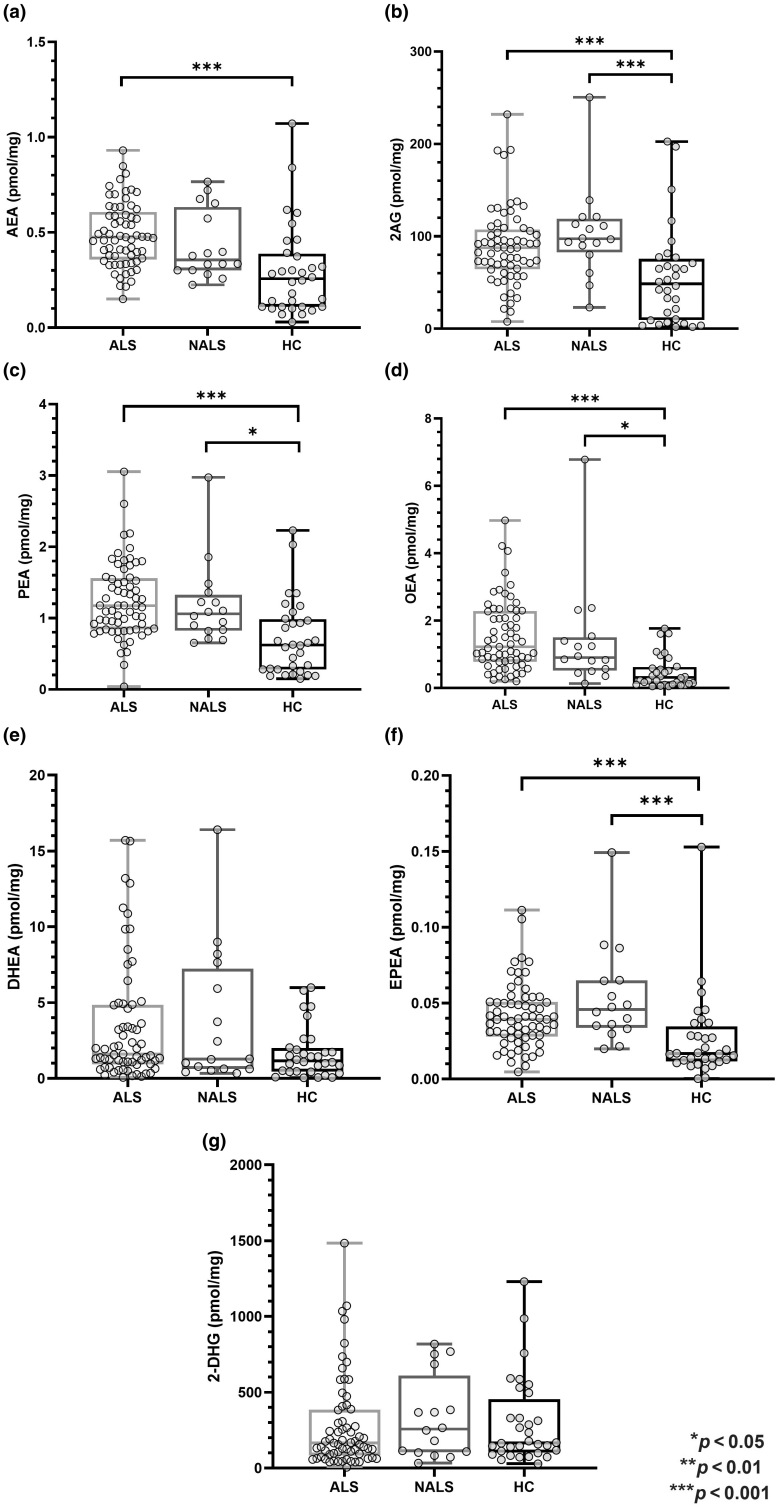
Comparison of circulating eCBome mediator levels amongst the three groups: ALS, NALS and HCs. Box‐and‐whisker dot plots comparing the levels of serum eCBome mediators (expressed as pmol/mg of lipid extract weight) between ALS, NALS and HC groups. Comparisons between the three groups were performed by means of the non‐parametric Kruskal–Wallis test, and post hoc comparisons by means of the Mann–Whitney *U* test using Bonferroni correction. *Significant *p* < 0.05; **significant *p* < 0.01; ***significant *p* < 0.001. 2‐AG, 2‐arachidonoylglycerol; 2‐DHG, 2‐docosahexaenoylglycerol; AEA, *N*‐arachidonoylethanolamine; ALS, amyotrophic lateral sclerosis; DHEA, docosahexaenoylethanolamide; eCBs, endocannabinoids; EPEA, eicosapentaenoylethanolamide; HC, healthy controls; NALS, neurological disease control; OEA, oleoylethanolamide; PEA, palmitoylethanolamide.

**FIGURE 2:**
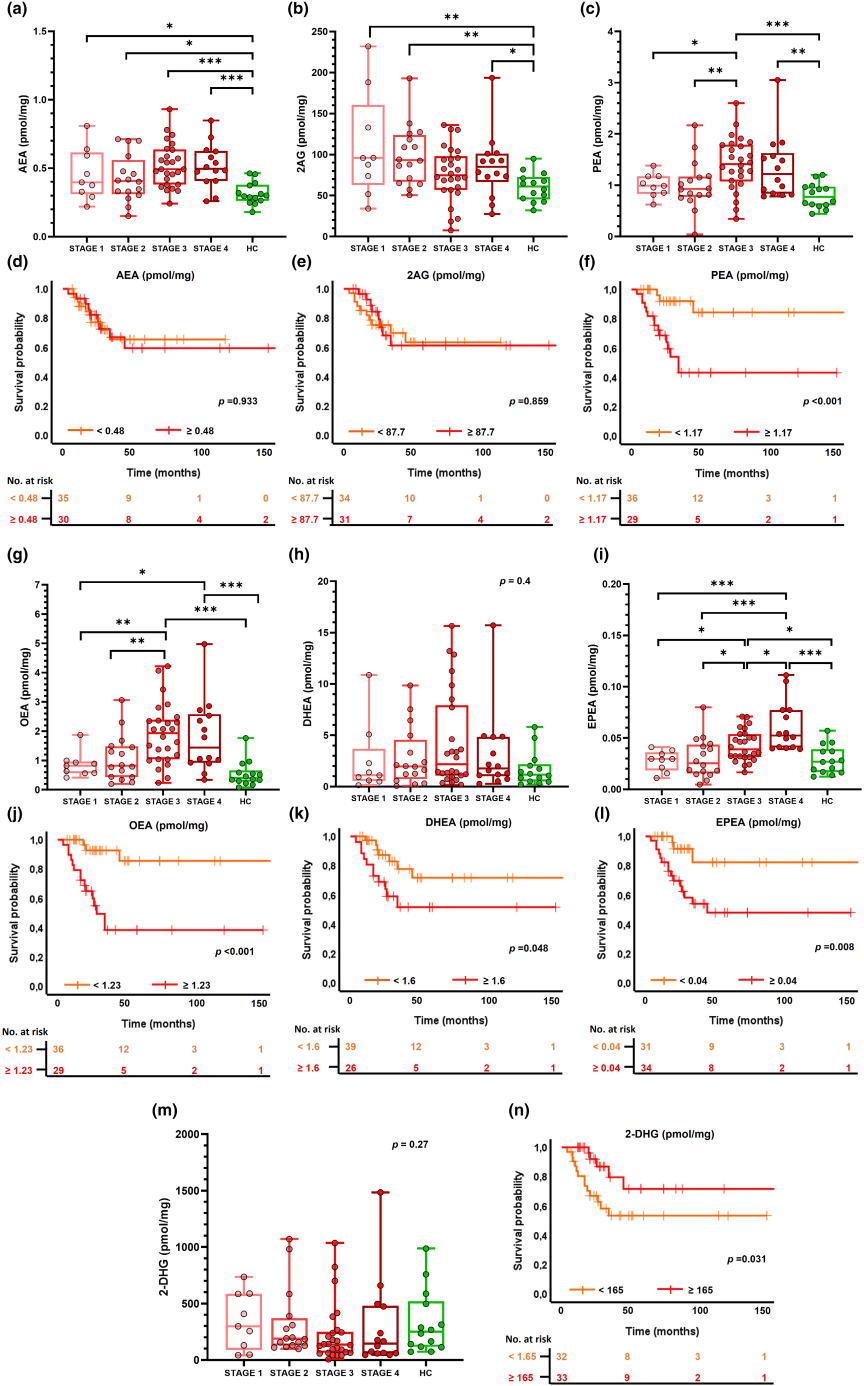
Circulating eCBome mediator levels across ALS clinical stages and their association with survival. Box‐and‐whisker dot plots of serum levels of eCBome mediators (expressed as pmol/mg of lipid extract weight) in healthy controls (HCs) and the ALS group divided according to King's clinical stages (Stage 1, Stage 2, Stage 3, Stage 4) and Kaplan–Meier curves of survival probability for AEA (a), (d), 2‐AG (b), (e), PEA (c), (f), OEA (g), (j), DHEA (h), (k), EPEA (i), (l) and 2‐DHG (m), (n). Group comparisons were performed only for the four ALS clinical stages by means of the non‐parametric Kruskal–Wallis test, and post hoc comparisons by means of the Mann–Whitney *U* test using Bonferroni correction. *Significant *p* < 0.05; **significant *p* < 0.01; ***significant *p* < 0.001. 2‐AG, 2‐arachidonoylglycerol; 2‐DHG, 2‐docosahexaenoylglycerol; AEA, *N*‐arachidonoylethanolamine; DHEA, docosahexaenoylethanolamide; EPEA, eicosapentaenoylethanolamide; OEA, oleoylethanolamide; PEA, palmitoylethanolamide.

The Kruskal–Wallis test disclosed a significant group effect for all investigated eCBome mediators except for 2‐docosahexaenoylglycerol (2‐DHG) (*p* = 0.547) and docosahexaenoylethanolamide (DHEA) (*p* = 0.092). Post hoc analysis revealed that ALS patients displayed significantly higher levels of AEA (*p* < 0.001), 2‐AG (*p* < 0.001), oleoylethanolamide (OEA) (*p* < 0.001), palmitoylethanolamide (PEA) (*p* < 0.001) and eicosapentaenoylethanolamide (EPEA) (*p* < 0.001) compared to HCs, but not NALS (all *p* > 0.374) (Figure 1). In addition, NALS patients showed significantly higher levels of eCBome mediators with respect to HCs for 2‐AG (*p* < 0.001), OEA (*p* = 0.022), PEA (*p* = 0.02) and EPEA (*p* < 0.001). Across ALS clinical stages and compared with HCs, circulating eCBome mediator levels were significantly different for AEA (*p* < 0.001), 2‐AG (*p* = 0.017), PEA (*p* < 0.001), OEA (*p* < 0.001) and EPEA (*p* < 0.001), but not for 2‐DHG (*p* = 0.265) and DHEA (*p* = 0.395). Interestingly, post hoc analysis revealed that HCs differed from the ALS group especially for patients belonging to more advanced clinical stages (i.e., 3 and 4) and with significantly higher values of circulating eCBome mediators (Figure 2). Finally, stratifying ALS patients based on the type of onset (bulbar vs. spinal), no significant differences for eCBome mediators were found (all *p* > 0.25) except for 2‐AG (*p* = 0.01) which was higher in the spinal phenotype (Data S1).

### Longitudinal analysis of circulating eCBs

**FIGURE 3:**
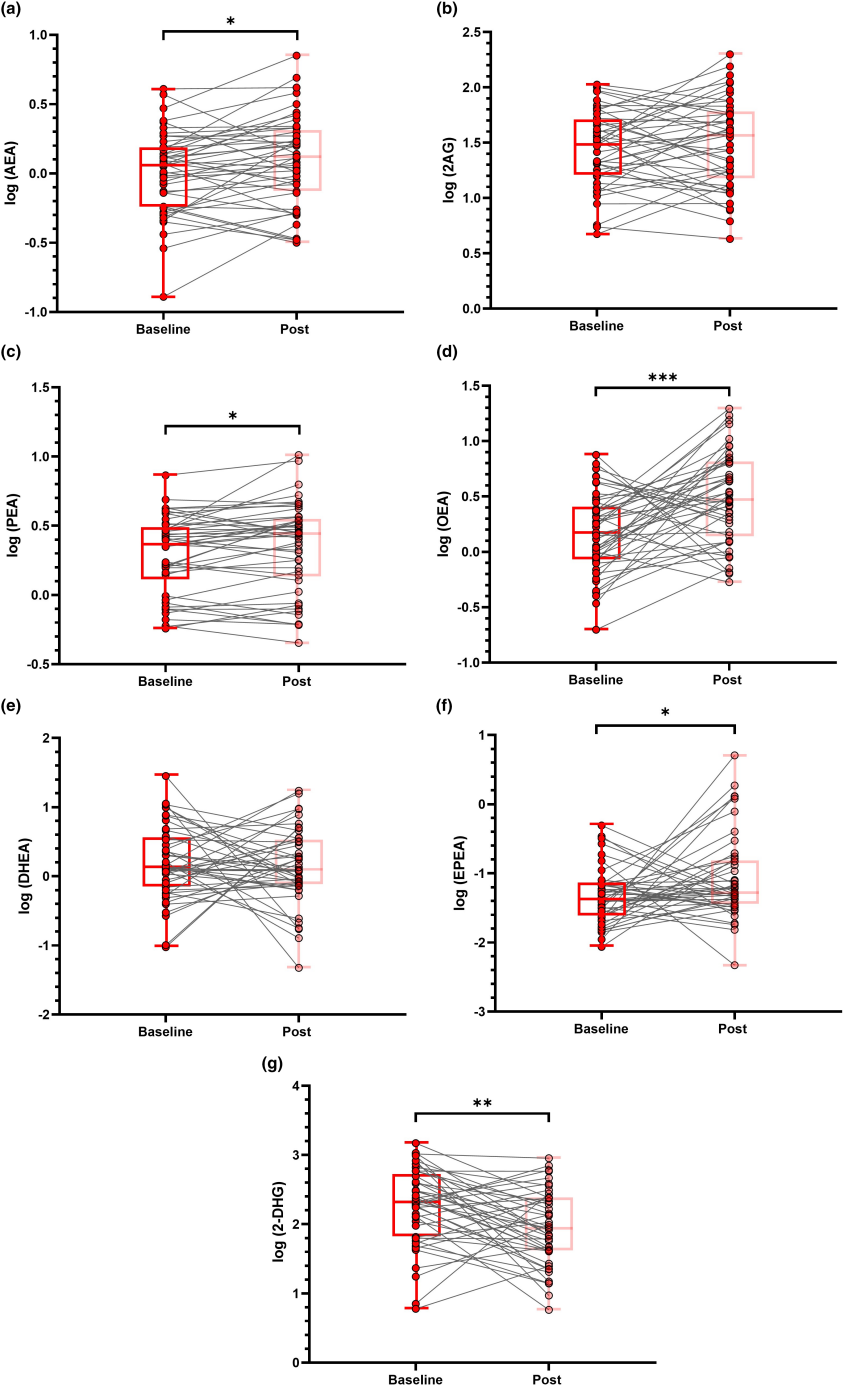
Longitudinal assessment of circulating eCBome mediators. Box‐and‐whisker dot plots of the log transformed values of AEA (a), 2‐AG (b), PEA (c), OEA (d), DHEA (e), EPEA (f), 2‐DHG (g) at baseline and follow‐up in 46 ALS patients. Comparisons of eCBome mediator levels over time were performed using three‐way repeated measures ANOVA, whilst post hoc comparisons were by means of the paired *t* test using Bonferroni correction. *Significant *p* < 0.05; **significant *p* < 0.01; ***significant *p* < 0.001. 2‐AG, 2‐arachidonoylglycerol; 2‐DHG, 2‐docosahexaenoylglycerol; AEA, *N*‐arachidonoylethanolamine; DHEA, docosahexaenoylethanolamide; EPEA, eicosapentaenoylethanolamide; OEA, oleoylethanolamide; PEA, palmitoylethanolamide.

To further demonstrate the change of eCBome mediators according to disease activity, 46 ALS patients underwent longitudinal sample collections with a median (IQR) follow‐up time of 4.5 (4) months. As expected, the three‐way ANOVA model yielded a significant effect on eCBome mediators (*F*_(6,264)_ = 764.5, *p* < 0.001), indicating a different behaviour of circulating levels. Interestingly, a significant interaction between eCBome mediator levels and time was also found (*F*_(6,264)_ = 6.8, *p* < 0.001), suggesting that specific circulating mediators underwent dynamic changes in their levels over time. Post hoc analysis revealed that AEA (*p* = 0.026), PEA (*p* = 0.023), EPEA (*p* = 0.027) and OEA (*p* < 0.001) significantly increased over time, whilst 2‐DHG significantly decreased (*p* = 0.003) (Figure 3). No significant change was observed for DHEA (*p* = 0.69) and 2‐AG (*p* = 0.39).

Moreover, a significant triple interaction of eCBome mediators, time and ALS Functional Rating Scale Revised median monthly decline (ΔALSFRS‐R) was also found (*F*_(6,264)_ = 2.1, *p* = 0.048), suggesting that ALS patients displaying a more aggressive disease (fast progressors) showed greater alterations of eCBome mediator levels over time than slow progressor patients. Post hoc analysis revealed that this effect was more pronounced for OEA levels (*p* = 0.048) and EPEA (*p* = 0.023), showing increased levels in more aggressive patients at follow‐up. No significant effect was observed for the remaining mediators (all *p* > 0.3). Similar results were obtained by performing a correlation analysis between ΔALSFRS‐R and the change over time of each mediator, being statistically significant for EPEA (*r* = 0.368, *p* = 0.012) and OEA (*r* = 0.295, *p* = 0.047).

### Association of circulating eCBome mediators with survival

Based on the association between eCBome mediator levels and disease activity, the prognostic performance of these biomarkers was also evaluated. The association of clinical and eCBome mediator levels with survival is summarized in Table 2. Specifically, it was found that clinical factors such as older age at onset, bulbar onset, a faster decline of disease disability, a shorter diagnostic delay and more advanced King's stage were associated with shorter survival. Interestingly, eCBome mediator levels such as a higher value of PEA, EPEA and OEA and a lower value of 2‐DHG were also associated with a poor outcome. In the multivariate analysis, four variables (older age, faster decline of disease disability and higher levels of OEA and PEA) survived as independent determinants of shorter survival.

**TABLE 2:** Multivariate analysis of survival predictors.

Variable	Crude HR (95% CI)	*p* value	Adjusted HR (95% CI)	*p* value
Clinical covariates				
Age at onset	1.05 (1.01, 1.1)	**0.021**	1.05 (1.01, 1.1)	**0.009**
Sex (female)	1.31 (0.51, 3.38)	0.58		
Site of onset (bulbar)	4.21 (1.47, 12.03)	**0.007**	1.54 (0.32, 7.28)	0.59
ALSFRS‐R (total score)	0.96 (0.91, 1.01)	0.09		
Diagnostic delay	0.89 (0.81, 0.97)	**0.009**	0.92 (0.83, 1.03)	0.138
ΔALSFRS‐R (median points/month)	3.59 (2.05, 6.27)	**<0.001**	2.49 (1.32, 4.73)	**0.005**
FVC (%)	0.98 (0.97, 1.00)	0.1		
Cognition (impaired)	1.54 (0.6, 3.98)	0.37		
BMI (kg/m^2^)	0.92 (0.8, 1.06)	0.27		
King's stages	1.94 (1.08, 3.5)	**0.027**	1.47 (0.54, 4.01)	0.448
eCBome mediator covariates				
AEA (≥0.48 pmol/mg)	1.04 (0.41, 2.62)	0.93		
2‐AG (≥87.7 pmol/mg)	0.92 (0.36, 2.33)	0.86		
PEA (≥1.17 pmol/mg)	5.1 (1.67, 15.57)	**0.004**	0.03 (0.00, 0.52)	**0.017**
OEA (≥1.23 pmol/mg)	7.49 (2.16, 25.97)	**0.002**	77.14 (4.04, 1473.2)	**0.004**
DHEA (≥1.6 pmol/mg)	2.62 (1.01, 6.78)	0.05		
EPEA (≥0.04 pmol/mg)	5.57 (1.32, 15.81)	**0.016**	2.13 (0.36, 12.63)	0.406
2‐DHG (≥165 pmol/mg)	0.34 (0.12, 0.96)	**0.04**	0.42 (0.12, 1.45)	0.168

*Note*: In bold significant *p* < 0.05.

Abbreviations: 2‐AG, 2‐arachidonoylglycerol; 2‐DHG, 2‐docosahexaenoyl‐glycerol; AEA, *N*‐arachidonoylethanolamine; ALS, amyotrophic lateral sclerosis; ALSFRS‐R, ALS Functional Rating Scale Revised; ΔALSFRS‐R, ALSFRS‐R median monthly decline; BMI, body mass index; CI, confidence interval; DHEA, docosahexaenoyl ethanolamide; eCBs, endocannabinoids; EPEA, eicosapentaenoyl ethanolamide; F, female; HR, hazard ratio; M, male; FVC, forced vital capacity; OEA, oleoylethanolamine; PEA, palmitoylethanolamine.

Kaplan–Meier curves of survival probability confirmed that higher levels of PEA (*p* < 0.001), OEA (*p* < 0.001), EPEA (*p* = 0.008) and lower levels of 2‐DHG (*p* = 0.031) at recruitment were prognostic factors of poor outcome (Figure 2f,j,l,n). A borderline significant result was also observed for higher DHEA levels (Figure 2k).

### Cluster analysis

The four circulating eCBome mediators (i.e., PEA, EPEA, OEA and 2‐DHG) associated with poor outcomes were included in a cluster analysis to identify which eCBome profiles were associated with specific ALS clinical features.

**FIGURE 4:**
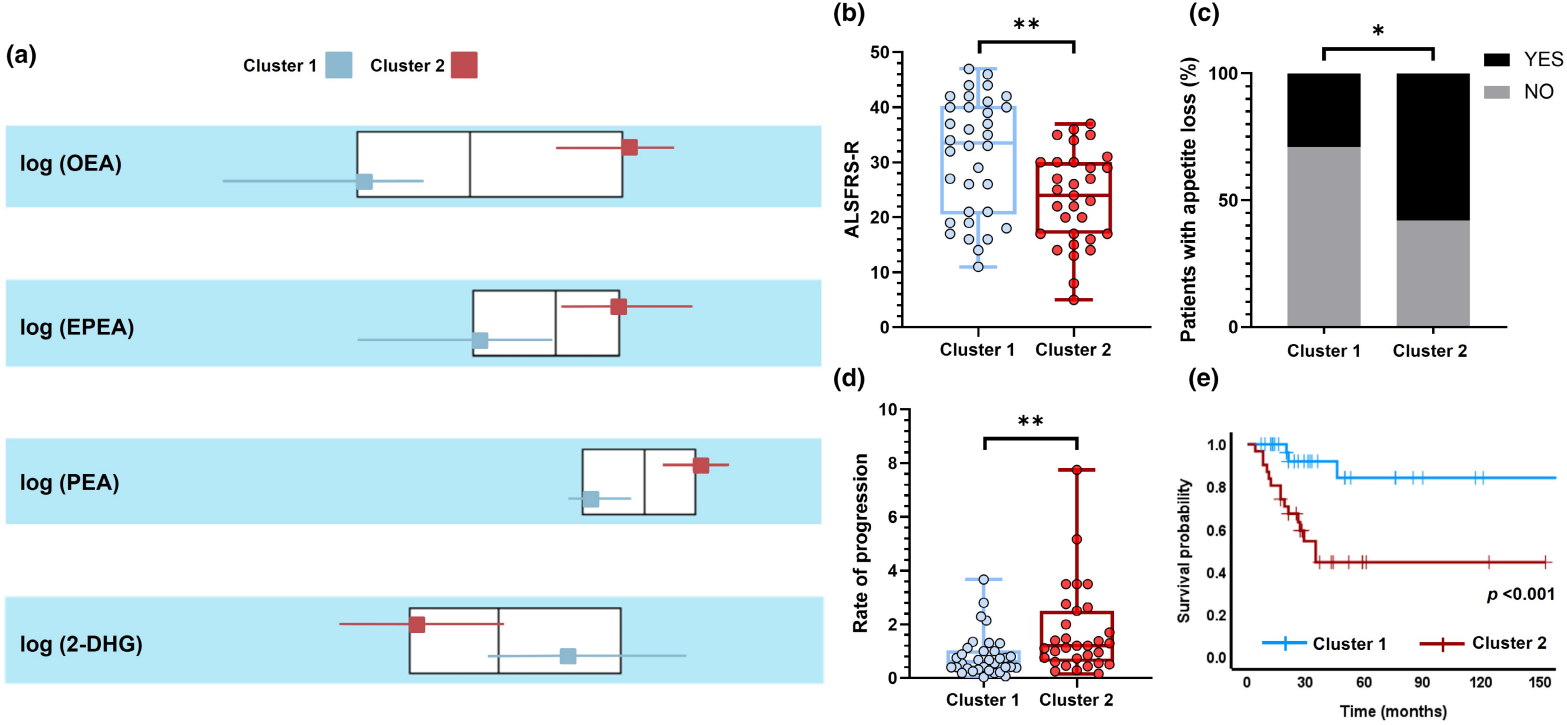
Cluster analysis of circulating eCBome mediators and clinical correlates of cluster membership. (a) Two‐step cluster analysis showing cluster separation between cluster 1 (light blue) and cluster 2 (red) for each mediator, represented by boxplots with medians and interquartile ranges. Comparison between the two clusters for the main clinical characteristics: motor disability indexed by the ALSFRS‐R scale (b), presence/absence of loss of appetite (c), rate of progression indexed by the ALSFRS‐R rate (d) and Kaplan–Meier curve of survival probability (e). *Significant *p* < 0.05; **significant *p* < 0.01. Note that patients belonging to cluster 2 exhibit a specific eCBome mediator profile with higher values of OEA, EPEA, PEA and lower level of 2‐DHG compared with cluster 1, and an overall more severe disease.

Two‐step cluster analysis identified two distinct clusters of mediator distribution, 47.7% of patients (*n* = 31) segregating to cluster 1 and 52.3% (*n* = 34) to cluster 2. The silhouette coefficient of 0.55 indicates reasonable cohesion and separation according to Kaufman and Rousseeuw [27]. Variable ranking revealed that eCBs that best predicted cluster memberships were OEA (1.00), EPEA (0.55), PEA (0.45), 2‐DHG (0.41) in descending order of importance, with distinct circulating levels between the two clusters (Figure 4a).

Based on allocated cluster membership, the demographic and clinical profiles of patients belonging to the two clusters were compared and it was found that patients with cluster 2 exhibited a more severe disease characterized by a more rapid disease course, shorter survival time with appetite loss than patients belonging to cluster 1, and the former cluster was characterized by higher levels of OEA, EPEA and PEA and lower level of 2‐DHG compared with cluster 1 (Figure 4a). A detailed comparison of demographic and clinical characteristics between the two clusters is available in Figure 4 and Data S1. As an ancillary analysis, an additional Kruskal–Wallis test was also run comparing the levels of eCBome mediators between the two ALS clusters, HCs and NALS. This analysis yielded a significant group effect for AEA (*p* < 0.001), 2‐AG (*p* < 0.001), OEA (*p* < 0.001), EPEA (*p* < 0.001), PEA (*p* < 0.001) and 2‐DHG (*p* < 0.001), but not for DHEA (*p* = 0.172). Post hoc analysis revealed that ALS patients belonging to the ‘severe’ cluster 2 displayed a unique eCB profile characterized by significantly higher levels of OEA and PEA and lower levels of 2‐DHG compared with HCs, NALS and the ‘mild’ cluster 1 (all *p* < 0.027) (Data S1). Interestingly, considering the five ALS patients carrying genetic mutations, it was found that only the *SOD1* patient showed an eCBome profile that fell within cluster 2 and corresponded to a severe phenotype (Data S1).

Finally, since some eCBome mediators (e.g., AEA and OEA) have important effects on eating behaviour, regulating food intake or satiety, ALS patients were stratified based on the presence of alterations in feeding behaviour such as overeating and loss of appetite. Interestingly it was found that patients with appetite loss had a selective increase in OEA levels (*p* = 0.013), whilst patients with overeating problems showed no difference in circulating eCB and congener levels compared with the ALS group without overeating (all *p* > 0.115) (see also Data S1).

## DISCUSSION

This study extends to human patients the results previously described in mouse models of ALS, where elevated levels of AEA and 2‐AG were found in the spinal cord of *SOD1* transgenic mice [11, 12, 13]. Additionally, the spectrum of alterations to anandamide derivatives (i.e., PEA and OEA) and the emerging omega‐3 eCB‐like molecules (i.e., EPEA and 2‐DHG) was expanded. Interestingly, cannabinoid receptors, such as CB_2_, have been shown to undergo a major upregulatory response in the spinal cord of mouse models of ALS at symptomatic stages, including *SOD1* mutants [14, 28] and TDP‐43 transgenic mice [15, 29]. This upregulation was also observed in postmortem tissues of ALS patients, including spinal cords [30] and primary motor cortex [31]. However, a recent study, also conducted in mutant *SOD1* (G93A) mice, revealed that the effects of CB_2_ receptor agonists in experimental ALS could depend on the stage of the disease, being detrimental in presymptomatic mice but changing to beneficial in later stages [32]. This opposite effect may depend on astrocyte‐dependent activation of microglial cells in the different ALS stages [32].

Interestingly, in the study cited above [28] the authors showed also that the daily administration of a selective CB_2_ agonist increased survival by inhibiting ALS‐associated neuroinflammation. Moreover, other studies showed that the genetic ablation of CB_2_ or its pharmacological blockade with selective antagonists worsened the disease progression [29]. All together, these discoveries seem to suggest that the eCB system exerts a protective role against the insult affecting the motor neurons.

Change in circulating levels of eCBs and congener molecules according to disease progression and survival has been demonstrated in our study in human ALS patients. Notably, our eCBome alterations are much stronger than those observed by a recent study [17], probably linked to several factors. For example, more ALS patients (65 vs. 41) were recruited with an overall more severe disease (ALSFRS‐R 27 vs. 37.5), more metabolites (7 vs. 5) were analysed and finally HCs and NALS matched for sex and age of the ALS population were included. Furthermore, although the analytical methodologies are quite comparable, in our study specific deuterated internal standards were used for each analyte, which provided greater accuracy and specificity in quantification.

In view of the well‐recognized anti‐inflammatory role of these molecules, it is believed that the altered levels of circulating eCBome mediator at different disease stages may be part of a compensatory mechanism of the body to halt the massive release of cytokines and thus the hyperactivated inflammatory response that is known to exacerbate the severity of ALS and be crucial to the prognosis [8].

In this regard, OEA and, especially, PEA are known to suppress inflammation through mechanisms depending on peroxisome proliferator‐activated receptor α (PPAR‐α) and nuclear factor‐κB activation [33]. DHEA (also known as synaptamide) has also been shown recently to produce both neurogenic and anti‐inflammatory effects in vitro and in vivo [34, 35]. Additionally, PEA has been shown to improve respiratory symptoms and be an adjunctive treatment to riluzole in ALS patients [36]. Interestingly, the prognostic values of circulating eCBome mediators have also been outlined in patients with end‐stage renal disease, where increasing serum OEA and PEA were associated with higher mortality risk and higher levels of proinflammatory cytokines such as interleukin 6 [37]. Regarding the role of EPEA and 2‐DHG, the mechanisms that regulate their levels are much less known. The few studies available demonstrate that EPEA exerts anti‐proliferative and anti‐cancer effects [38], also counteracting inflammation [39] and playing a role in ageing [40]. Noteworthy, a recent study in a cohort of ALS patients showed that eicosapentaenoic acid (EPA), a precursor of EPEA, is associated with a lower risk of poor prognosis [41]. Conversely, in *SOD1* mutant mice, EPA induced a significantly shorter lifespan, probably due to an accumulation of omega‐3 fatty acid oxidation products, thus casting some doubts on the benefit of dietary omega‐3 fatty acid supplements in ALS patients [42].

On the other hand, altered eCBome mediator levels may be the cause of a maladaptive mechanism which contributes to the early onset of symptoms and disease progression. This hypothesis was the main conclusion of a study conducted in *SOD1* mutant mice in early presymptomatic phases in which a down‐regulatory response of CB_1_ receptors was associated with the increase in glutamate receptors [43]. Given the role of CB_1_ receptors in the control of glutamate homeostasis, the reduction in these receptors found in *SOD1* mutant mice may predispose motor neurons to excitotoxic events, and then contribute to pathogenesis. Similar conclusions come from the study of Rossi et al. who attributed changes in the CB_1_ receptor sensitivity in ALS to the motoneuronal damage caused by excitotoxicity [44]. Yet, eCBs and their congeners, being metabolized by eicosanoid biosynthetic enzymes such as cyclooxygenase‐2, lipoxygenases and cytochrome P450, could lead to the production of secondary metabolites with uncontrolled effects [45]. These latter metabolites might, directly or indirectly, act as mediators of oxidative stress, inflammation and in the regulation of neuronal hyperexcitability [46] in ALS. However, even without considering the still controversial role of CB1R and CB2R and their endogenous agonists (i.e., AEA and 2‐AG) in ALS, it is believed that, in view of the widely documented protective effects of PEA, OEA, EPEA and DHEA via non‐cannabinoid receptors [47], most of the changes observed here represent an attempt to reduce the inflammation and neuronal damage typical of later stages of this disorder.

Nevertheless, cluster analysis of circulating eCBome mediators revealed two distinct biochemical profiles associated with distinct disease characteristics. Patients that were grouped into cluster 1 (‘mild’) appeared to have few observed differences compared to HCs and NALS. Conversely, patients grouped in cluster 2 (‘severe’) had significant eCBome mediator abnormalities, with a divergent profile for OEA/PEA (increase) and 2‐DHG levels (decrease). Interestingly, the dramatically altered eCBome in cluster 2 patients was associated with respiratory impairment, a higher percentage of patients belonging to advanced disease stages, a faster disease progression rate, significantly shorter survival, and a higher proportion of patients with loss of appetite, typical of this cluster. The latter feature is particularly interesting in view of the well‐established role of eCBs, in particular OEA, in regulating feeding behaviour and body weight. Specifically, administration of OEA to mice reduces food intake and body weight gain, probably through PPAR‐α in the gastrointestinal tract [48] and/or through actions in the area postrema [49]. A study of individuals with chronic vomiting syndrome found that circulating concentrations of OEA were significantly higher in the vomiting (sick) phase compared to the well phase and were strongly correlated with poor sleep [50]. These data, together with their function to promote satiety, suggest that OEA concentrations may contribute to anorexia and reduced sleep quality and thereby contribute to disease severity. Given that loss of appetite is reported by approximately half of ALS patients and is related to weight loss and a worse prognosis [6], it is believed that OEA could be considered as a potential biochemical signature of this clinical feature in ALS patients.

However, despite the novelty, some key limitations of this study must be considered. These include the lack of longitudinal data regarding the levels of eCBome mediators in the HC and NALS groups as well as the lack of information on patients' dietary habits via validated nutritional questionnaires. Future studies are necessary to clarify these aspects and to shed light on the molecular mechanism(s) causing the eCBome dysregulation and its role in ALS. Furthermore, the potential value of specific changes related to one or more eCBome components as diagnostic and/or prognostic marker of the disease is a not less important point worth exploring in the future.

## CONCLUSION

In conclusion, our study demonstrates the alteration of circulating eCB and related mediator levels in ALS patients at different stages of the disease and over time, as well as their potential prognostic value; however, the level of these mediators does not appear to be disease specific.

Interestingly, the existence of a specific clinical subtype of ALS was also demonstrated characterized by more aggressive disease and loss of appetite and associated with a unique eCBome mediator profile, which also differed from the NALS group. Therefore, this study lays the grounds for the use of eCBs and eCB‐like metabolites both as biomarkers of disease activity and, hopefully, for therapeutic purposes.

## AUTHOR CONTRIBUTIONS

**Raffaele Dubbioso:** Conceptualization; methodology; data curation; supervision; formal analysis; investigation; writing – original draft; writing – review and editing. **Fabio Arturo Iannotti:** Methodology; validation; formal analysis. **Gianmaria Senerchia:** Project administration; supervision; formal analysis. **Roberta Verde:** Conceptualization; visualization; resources. **Valentina Virginia Iuzzolino:** Methodology; validation; visualization. **Myriam Spisto:** Conceptualization; investigation; project administration. **Ines Fasolino:** Methodology; validation; investigation. **Fiore Manganelli:** Conceptualization; supervision. **Vincenzo Di Marzo:** Conceptualization; supervision; writing – review and editing; writing – original draft. **Fabiana Piscitelli:** Writing – original draft; writing – review and editing; conceptualization; methodology; software; formal analysis; data curation; supervision; investigation.

## FUNDING INFORMATION

This work is supported by Next Generation EU (NGEU) and by the Ministry of University and Research (MUR), National Recovery and Resilience Plan (NRRP), through the projects INFLAMM‐ALS (Progetti di Ricerca di Rilevante Interesse Nazionale, PRIN2022, CUP: E53D23011330006) and SENSATION‐ALS (PRIN‐PNRR2022, CUP: E53D23019760001) funded by the European Union—Next Generation EU.

## CONFLICT OF INTEREST STATEMENT

The authors declare that they have no competing interests.

## ETHICS STATEMENT

A written informed consent was obtained from all subjects according to the Declaration of Helsinki before enrolment in the study. The study protocol was approved by the local Ethics Committee (protocol number 100/17/ES01 and 151/2023).

## Supporting information


Data S1.

## Data Availability

The dataset supporting the conclusions of the paper will be made available by the authors, to any qualified researcher, without breaching participant confidentiality.
