# Supplementary material for: Circulating endocannabinoidome signatures of disease activity in amyotrophic lateral sclerosis
Source: Eur J Neurol. 2024 Aug 16;31(10):e16400. doi: 10.1111/ene.16400 (PMC11414802; doi:10.1111/ene.16400)
Supplement: Supplementary file 1 — Data S1. [file ENE-31-e16400-s001.docx]

**Article title:** “Circulating endocannabinoidome signatures of disease activity in amyotrophic lateral sclerosis”

**Author names:** Raffaele Dubbioso^1†^, Fabio Arturo Iannotti^2^, Gianmaria Senerchia^1^, Roberta Verde^2^, Valentina Virginia Iuzzolino^1^, Myriam Spisto^1^, Ines Fasolino^3^, Fiore Manganelli^1^, Vincenzo Di Marzo^2, 4^, Fabiana Piscitelli^2†^.

**Journal name:** *European Journal of Neurology*

^†^**Correspondence to:
Raffaele Dubbioso**

Department of Neurosciences, Reproductive Sciences and Odontostomatology, University of Naples Federico II, Via Sergio Pansini, 5, 80131 Naples, Italy

Tel: +390817464587

Email: raffaele.dubbioso@unina.it

**Fabiana Piscitelli**

Institute of Biomolecular Chemistry - National Research Council (ICB-CNR),Via Campi Flegrei, 34, 80078 Pozzuoli (NA), Italy

Tel: +390818675309
Email: [fpiscitelli@icb.cnr.it](mailto:fpiscitelli@icb.cnr.it)

# Material and methods

**Participants and clinical characterisation**

Patients with ALS met a diagnosis of “probable”, “probable laboratory-supported” or “definite” ALS, as per the revised El Escorial criteria (Brooks et al., 2000). Disease severity and clinical staging were assessed by the revised ALS Functional Rating Scale (ALSFRS-R) (Cedarbaum et al., 1999) and by ALS King’s scale (Roche et al., 2012), respectively. The ALSFRS-R was also used to compute the disease progression rate (Kimura et al., 2006)by applying the following formula: ((ALSFRS-R_rate: (48 - ALSFRS-R at the study inclusion)/(disease duration in months)). In addition, muscle strength was assessed by Medical Research Council (MRC) scale in each body region (Dubbioso et al., 2023); and the upper motor neuron (UMN) burden by means of the Penn Upper Motor Neuron Score (PUMNS) (Quinn et al., 2020). Height and weight were also measured to calculate the body mass index (BMI).

Respiratory function was assessed through spirometry with the patient sitting upright. Results for forced vital capacity (FVC) were expressed as a percentage of predicted value, from an average of three trials (Czaplinski, 2005).

Lastly, neuropsychological tests were performed to classify patients according to consensus criteria (Strong et al., 2017) as having “normal cognition” (i.e., ALS-nc) or “cognitive and/or behavioural impairment” (i.e., ALS with cognitive impairment, ALS-ci; ALS with combined cognitive and behavioural impairment, ALS-cbi; ALS with behavioural impairment, ALS-bi). A detailed neuropsychological battery has been described elsewhere (De Lucia et al., 2020; Moretta et al., 2022).

We excluded participants if they had (1) acute or chronic infections or autoimmune disorders; (2) kidney or chronic liver diseases or regular alcohol intake (over 80 g/day for 6 months).

Since circulating eCBs are important regulators of eating behaviours in terms of food intake or satiety, we also classified ALS patients as having or not eating disturbances by using the specific items of the Mild Behavioural Impairment Checklist (MBI-C) (Ferraro et al., 2023). Specifically, item 20 is related to changes in eating behavior (e.g. overeating, cramming the mouth), while item 21 is related to loss of appetite, asking whether the patient no longer finds food tasty/ enjoyable or is eating less. For both questions we reported whether the disorder was present or not.

**Endocannabinoid and related molecules analysis.**

Serum was collected in 7 mL clot activator tubes (BD Vacutainer®) at inclusion and centrifuged at 1500 g for 10 min at room temperature. Aliquots (from 1 to 2 mL) were stored at −80°C until use. Times between blood collection and centrifugation were always shorter than 30 min. Serum (200 µl) was sonicated and extracted with chloroform/methanol (2 : 1 , vol/vol) containing internal standards for AEA, 2AG, PEA, OEA, docosahexaenoyl ethanolamide (DHEA), eicosapentaenoyl ethanolamide (EPEA) and 2-docosahexaenoyl glycerol (2-DHG) ([^2^H]_8_ AEA 5 pmol; [^2^H]_5_ 2AG, [^2^H]_4_ PEA, [^2^H]_2_ OEA, 2-heptadecanoyl glycerol, for 2-DHG quantification, 50 pmol each; [^2^H]_4_ DHEA and [^2^H]_4_ EPEA 10 pmol each). The lipid-containing organic phase was dried down, weighed, and dissolved in 100 µl of methanol for eCB and related molecules quantification by LC-APCI-MS (LCMS-2020, Shimadzu) as previously reported (Ferrara et al., 2019; Piscitelli et al., 2020; Zamberletti et al., 2017)**.**

**Statistical Analysis**

Specifically, we compared demographic and endocannabinoids levels among the three groups of participants (ALS vs HC vs NALS), between ALS patients with bulbar onset and spinal onset, and across the ALS clinical stages by means of χ^2^ test (categorical variables) or the Kruskal–Wallis test (continuous variables), the post-hoc comparisons between groups were performed by means of Mann-Whitney U test.

Univariate and multivariate analysis for survival (calculated from onset to death or censoring date) was performed with the Cox proportional hazards model, and p values were computed using Wald test. In the Cox model we included the following variables: age, sex, disease onset (spinal vs bulbar), diagnostic delay, disease progression rate (ALSFRS-R_rate), baseline ALSFRS-R, baseline FVC (%), BMI, cognitive and/or behavioural impairment (yes or no), Kings stages, and eCBs levels stratified according to the median value.

Survival curves were constructed with the Kaplan–Meier method only for the eCBs covariates that were associated with survival time according to Cox regression analysis, namely EPEA, PEA, OEA, and 2-DHG. Log-rank test was then applied to compute the differences between the curves.

Circulating eCBs were also included in the longitudinal analysis, after log transformation of raw values, by applying a three-way repeated measure ANOVA, considering as within-subject factors: *time* (baseline vs Post) and *eCBs levels* (logAEA, log2AG, logEPEA, logPEA, logOEA, logDHEA, log2-DHG), and between-subject factor *ΔALSFRS-*R (slow progressor vs fast progressor). This last factor was defined by stratifying ALS patients based on the monthly ALSFRS-R points lost during the follow-up time, if it was ≥ 1 the patient was defined as fast progressor, otherwise if it was < 1 as slow progressor. As an additional analysis, we also performed a Pearson correlation analysis between the ΔALSFRS-R and the change in each mediator over time expressed as the difference between the follow-up value compared to the baseline value (ΔeCB).

Finally, the levels of eCBs were included in a 2-step cluster analysis using Likelihood distance measure. The number of clusters was not fixed a priori, and the Bayesian Information Criterion (BIC) was used to determine the number of clusters. Based on cluster membership of individual ALS patients, cluster sizes were determined, and silhouette analyses run using the STATS CLUS SIL extension of SPSS. The hierarchy of input variables was calculated to rank predictor importance, i.e. the levels of which eCBs best segregate the patients. Cluster membership was then visualised by box plots with interquartile intervals along the most relevant eCBs to demonstrate case separation. In post hoc analyses, the clinical profiles of the clusters identified were contrasted. Statistical analyses were carried out using the SPSS 29.0 statistical package (SPSS, Chicago, IL, USA). Alpha inflation due to multiple comparisons was controlled using Bonferroni correction when appropriate.

**References**

Brooks, B. R., Miller, R. G., Swash, M., & Munsat, T. L. (2000). El Escorial revisited: revised criteria for the diagnosis of ALS. *Amyotrophic Lateral Sclerosis and Other Motor Neuron Disorders : Official Publication of the World Federation of Neurology, Research Group on Motor Neuron Diseases*, *1*(5), 293–299. http://www.ncbi.nlm.nih.gov/pubmed/11464847

Cedarbaum, J. M., Stambler, N., Malta, E., Fuller, C., Hilt, D., Thurmond, B., & Nakanishi, A. (1999). The ALSFRS-R: a revised ALS functional rating scale that incorporates assessments of respiratory function. *Journal of the Neurological Sciences*, *169*(1–2), 13–21. https://doi.org/10.1016/S0022-510X(99)00210-5

Czaplinski, A. (2005). Forced vital capacity (FVC) as an indicator of survival and disease progression in an ALS clinic population. *Journal of Neurology, Neurosurgery & Psychiatry*, *77*(3), 390–392. https://doi.org/10.1136/jnnp.2005.072660

De Lucia, N., Ausiello, F. P., Spisto, M., Manganelli, F., Salvatore, E., & Dubbioso, R. (2020). The emotional impact of COVID-19 outbreak in amyotrophic lateral sclerosis patients: evaluation of depression, anxiety and interoceptive awareness. *Neurological Sciences*, *41*(9), 2339–2341. https://doi.org/10.1007/s10072-020-04592-2

Dubbioso, R., Spisto, M., Hausdorff, J. M., Aceto, G., Iuzzolino, V. V., Senerchia, G., De Marco, S., Marcuccio, L., Femiano, C., Iodice, R., Salvatore, E., Santangelo, G., Trojano, L., & Moretta, P. (2023). Cognitive impairment is associated with gait variability and fall risk in amyotrophic lateral sclerosis. *European Journal of Neurology*, *30*(10), 3056–3067. https://doi.org/10.1111/ene.15936

Ferrara, A. L., Piscitelli, F., Petraroli, A., Parente, R., Galdiero, M. R., Varricchi, G., Marone, G., Triggiani, M., Di Marzo, V., & Loffredo, S. (2019). Altered Metabolism of Phospholipases, Diacylglycerols, Endocannabinoids, and *N* -Acylethanolamines in Patients with Mastocytosis. *Journal of Immunology Research*, *2019*, 1–14. https://doi.org/10.1155/2019/5836476

Ferraro, P. M., Gervino, E., De Maria, E., Meo, G., Ponzano, M., Pardini, M., Signori, A., Schenone, A., Roccatagliata, L., & Caponnetto, C. (2023). Mild behavioral impairment as a potential marker of predementia risk states in motor neuron diseases. *European Journal of Neurology*, *30*(1), 47–56. https://doi.org/10.1111/ene.15570

Kimura, F., Fujimura, C., Ishida, S., Nakajima, H., Furutama, D., Uehara, H., Shinoda, K., Sugino, M., & Hanafusa, T. (2006). Progression rate of ALSFRS-R at time of diagnosis predicts survival time in ALS. *Neurology*, *66*(2), 265–267. https://doi.org/10.1212/01.wnl.0000194316.91908.8a

Moretta, P., Spisto, M., Ausiello, F. P., Iodice, R., De Lucia, N., Santangelo, G., Trojano, L., Salvatore, E., & Dubbioso, R. (2022). Alteration of interoceptive sensitivity: expanding the spectrum of behavioural disorders in amyotrophic lateral sclerosis. *Neurological Sciences*, *43*(9), 5403–5410. https://doi.org/10.1007/s10072-022-06231-4

Piscitelli, F., Guida, F., Luongo, L., Iannotti, F. A., Boccella, S., Verde, R., Lauritano, A., Imperatore, R., Smoum, R., Cristino, L., Lichtman, A. H., Parker, L. A., Mechoulam, R., Maione, S., & Di Marzo, V. (2020). Protective Effects of *N* -Oleoylglycine in a Mouse Model of Mild Traumatic Brain Injury. *ACS Chemical Neuroscience*, *11*(8), 1117–1128. https://doi.org/10.1021/acschemneuro.9b00633

Quinn, C., Edmundson, C., Dahodwala, N., & Elman, L. (2020). Reliable and efficient scale to assess upper motor neuron disease burden in amyotrophic lateral sclerosis. *Muscle and Nerve*, *61*(4), 508–511. https://doi.org/10.1002/mus.26764

Roche, J. C., Rojas-Garcia, R., Scott, K. M., Scotton, W., Ellis, C. E., Burman, R., Wijesekera, L., Turner, M. R., Leigh, P. N., Shaw, C. E., & Al-Chalabi, A. (2012). A proposed staging system for amyotrophic lateral sclerosis. *Brain*, *135*(3), 847–852. https://doi.org/10.1093/brain/awr351

Strong, M. J., Abrahams, S., Goldstein, L. H., Woolley, S., Mclaughlin, P., Snowden, J., Mioshi, E., Roberts-South, A., Benatar, M., HortobáGyi, T., Rosenfeld, J., Silani, V., Ince, P. G., & Turner, M. R. (2017). Amyotrophic lateral sclerosis - frontotemporal spectrum disorder (ALS-FTSD): Revised diagnostic criteria. *Amyotrophic Lateral Sclerosis and Frontotemporal Degeneration*, *18*(3–4), 153–174. https://doi.org/10.1080/21678421.2016.1267768

Zamberletti, E., Piscitelli, F., De Castro, V., Murru, E., Gabaglio, M., Colucci, P., Fanali, C., Prini, P., Bisogno, T., Maccarrone, M., Campolongo, P., Banni, S., Rubino, T., & Parolaro, D. (2017). Lifelong imbalanced LA/ALA intake impairs emotional and cognitive behavior via changes in brain endocannabinoid system. *Journal of Lipid Research*, *58*(2), 301–316. https://doi.org/10.1194/jlr.M068387

**Table S1**

**Demographic data of neurological disease controls (NALS) group**

| **Diagnosis** | **Sex** | **Age (years)** | **disease duration (months)** |
| --- | --- | --- | --- |
| Hereditary motor neuropathy | M | 50 | 132 |
| Lower limb weakness without evidence of MND | M | 53 | 60 |
| Parkinson's Disease | M | 64 | 180 |
| Vascular Dementia | M | 79 | 36 |
| Conus Myelitis | M | 55 | 25 |
| Mitochondrial myopathy | M | 53 | 3 |
| Inclusion body myopathy (IBM) and fronto-temporal dementia (FTD) | M | 76 | 13 |
| Myalgia-cramp-fasciculation syndrome | M | 88 | 1 |
| Cerebrovascular disease with pseudobulbar syndrome without evidence of MND | F | 44 | 1 |
| Primary Lateral Sclerosis | F | 71 | 60 |
| Progressive external ophthalmoplegia (PEO) | F | 68 | 11 |
| Anterior spinal artery syndrome | F | 75 | 1 |
| Ischemic stroke with dysarthria and pseudobulbar syndrome without evidence of MND | F | 66 | 1 |
| Spinocerebellar ataxia (SCA) | F | 69 | 72 |
| Poliomyelitis | M | 73 | 840 |
| Pseudobulbar and cerebellar syndrome without evidence of MND | M | 70 | 1 |

**Table S2.**

**Demographic and clinical data of ALS patients stratified according to cluster analysis.**

|  | **CLUSTER 1** | **CLUSTER 2** | ***P* value** |
| --- | --- | --- | --- |
| **Age at testing, years** | 63.5 (14.5) | 70 (23.5) | 0.486 |
| **Sex (M/F)** | 20/14 | 21/10 | 0.457 |
| **Disease duration since onset, months** | 27.5 (32.3) | 27 (24) | 0.642 |
| **ALSFRS-R score** | 33.5 (19) | 24 (13) | **0.003** |
| **Disease progression rate (ALSFRS-R points lost/month)** | 0.6 (0.6) | 1.2 (1.6) | **0.003** |
| **% of patients belonging to King's stages 3 and 4 (advanced stages)** | 35% (12/34) | 90% (28/31) | **<0.001** |
| **Onset (spinal/bulbar)** | 29/5 | 23/8 | 0.264 |
| **% of patients with Cognitive and/or behavioural Impairment** | 41% (14/34) | 58% (18/31) | 0.174 |
| **FVC (%)** | 85.5 (43.8) | 49 (45) | **0.006** |
| **BMI (kg/m^2^)** | 23.4 (5.8) | 23.3 (4.3) | 0.58 |
| **PUMNS total score (max 32)** | 13.5 (8.8) | 15 (11) | 0.09 |
| **MRC total score (max 130)** | 101 (45.3) | 74 (59.5) | **0.009** |
| **% of patients with loss of appetite** | 29% (10/34) | 58% (18/31) | **0.02** |
| **% of patients with overeating** | 24% (8/34) | 36% (11/31) | 0.290 |
| **% of deceased patients** | 9% (3/34) | 48% (15/31) | **<0.001** |

Note: Values are expressed as median (interquartile range) or as percentage (frequencies). Comparisons between the two groups were performed by means of non-parametric Mann-Whitney U test; frequencies were compared by means of chi- square test. Values in bold type indicate significance p< 0.05

**Table S3.**

**Comparison of clinical data and circulating eCBome mediators between patients with bulbar and spinal onset**

|  | **Bulbar onset** | **Spinal Onset** | **p-value** |
| --- | --- | --- | --- |
| Number | 52 | 13 |  |
| Age at testing, years | 70.5 (19.3) | 65 (23) | 0.17 |
| Sex (M/F) | 3/16 | 5/8 | **0.04** |
| Disease duration since onset, months | 18 (14) | 29 (39) | **0.004** |
| ALSFRS-R score | 30 (15) | 27 (18) | 0.50 |
| FVC (%) | 70 (52) | 81 (77) | 0.23 |
| BMI (Kg/m2) | 22.1 (4.6) | 23.7 (5.4) | 0.14 |
| % of patients with loss of appetite | 61.5% | 38.5% | 0.13 |
| % of patients with overeating | 30.8% | 28.8% | 0.89 |
| King's stage I | 7.7% | 15.4% | 0.68 |
| King's stage II | 23.1% | 25% |  |
| King's stage III | 53.8% | 36.5% |  |
| King's stage IV | 15.4% | 23.1% |  |
| MRC total score (max 130), higher is better | 113.5 (31.5) | 84 (56) | **0.02** |
| PUMNS total score (max 32), lower is better | 16.5 (7.5) | 14 (10) | **0.04** |
| eCBome mediator |  |  |  |
| AEA (pmol/mg) | 0.40 (0.12) | 0.47 (0.29) | 0.62 |
| 2-AG (pmol/mg) | 68.54 (38.02) | 93.22 (42.77) | **0.01** |
| PEA (pmol/mg) | 1.09 (1.72) | 1.13 (0.62) | 0.79 |
| OEA (pmol/mg) | 1.3 (2.34) | 1.04 (1.24) | 0.41 |
| DHEA (pmol/mg) | 4.62 (9.11) | 1.39 (2.61) | 0.28 |
| EPEA (pmol/mg) | 0.032 (0.03) | 0.039 (0.03) | 0.25 |
| 2-DHG (pmol/mg) | 178.5 (450.7) | 161.4 (334.7) | 0.95 |

Note: Values are expressed as median (interquartile range). Comparisons between the two groups were performed by means of non-parametric Mann-Whitney U test. Values in bold type indicate significance p< 0.05.

**Table S4.**

**Comparison of circulating eCBome mediators between patients with/without loss of appetite and patients with/without overeating**

|  | **LOSS OF APPETITE** | | | **OVEREATING** | | |
| --- | --- | --- | --- | --- | --- | --- |
|  | **Yes** | **No** | ***P value*** | **Yes** | **No** | ***P value*** |
| **AEA (pmol/mg)** | 0.48 (0.25) | 0.46 (0.23) | 0.587 | 0.51 (0.26) | 0.47 (0.24) | 0.436 |
| **2AG (pmol/mg)** | 85.14 (34.73) | 87.65 (40.45) | 0.895 | 73.88 (30.64) | 92 (48.62) | 0.109 |
| **PEA (pmol/mg)** | 1.45 (0.86) | 1.09 (0.51) | 0.064 | 1.03 (0.42) | 1.18 (0.74) | 0.762 |
| **OEA (pmol/mg)** | 2.17 (2) | 1.02 (0.93) | **0.013** | 1.18 (1.02) | 1.26 (1.58) | 0.817 |
| **DHEA (pmol/mg)** | 2.18 (2.66) | 1.35 (4.19) | 0.308 | 1.26 (2.26) | 2.03 (3.79) | 0.145 |
| **EPEA (pmol/mg)** | 0.04 (0.02) | 0.04 (0.02) | 0.56 | 0.04 (0.03) | 0.04 (0.02) | 0.141 |
| **2-DHG (pmol/mg)** | 162.98 (311.19) | 165.41 (203.25) | 0.812 | 165.41 (265.37) | 162.98 (268.94) | 0.773 |

Note: Values are expressed as median (interquartile range). Comparisons between the two groups were performed by means of non-parametric Mann-Whitney U test. Values in bold type indicate significance p< 0.05.

**Figure S1**

**Comparison of circulating eCBome mediator levels among the four groups: ALS cluster 1, ALS cluster 2, NALS and HC.**


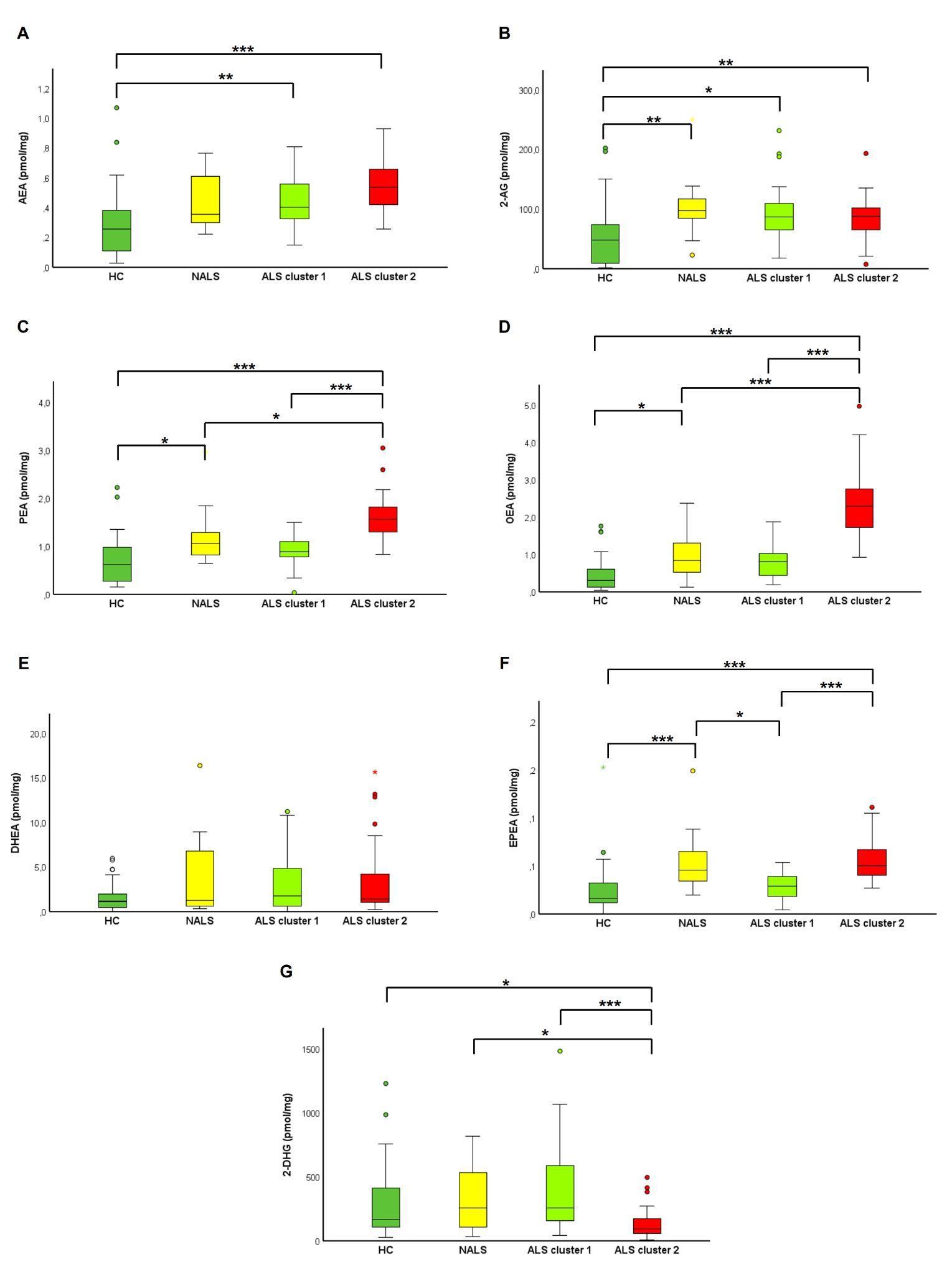


Comparisons among the four groups were performed by means of non-parametric Kruskal–Wallis test, while the post-hoc comparisons by means of Mann-Whitney U test using Bonferroni correction.

* Significant p < 0.05; ** significant p < 0.01; *** significant p < 0.001

**Figure S2**

**eCBome profile of the genetic cases and sporadic patients.**

**
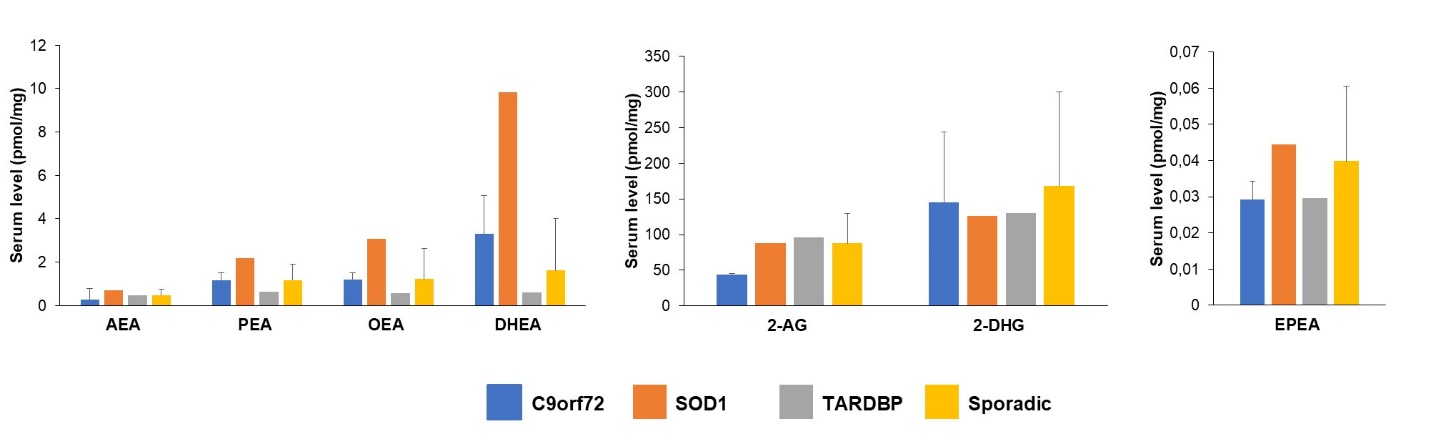
**

eCBome profile of the three patients carrying C9orf72 repeat expansion, one patient had mutations in the SOD1 gene and another in the TARDBP gene. Sporadic cases were ALS patients (N = 60) negative for the four most common ALS-causing genes (C9orf72, SOD1, TARDBP, and FUS). Data are expressed as median and IQR where possible.
